# Case Report of Herpes Zoster Ophthalmicus with Concurrent Parotitis

**DOI:** 10.21980/J8R93N

**Published:** 2023-04-30

**Authors:** Serena Tally, Michelle Brown, Edmund Hsu

**Affiliations:** * University of California, Irvine, Department of Emergency Medicine, Orange, CA

## Abstract

A 36-year-old immunocompetent female presented to the emergency department (ED) with five days of headache and left-sided facial pain. Physical exam showed conjunctival injection of the left eye with multiple vesicular lesions distributed along the V1 dermatome. Labs were remarkable for mild elevation in erythrocyte sedimentation rate (ESR) and c-reactive protein (CRP) with no elevation in white blood cell (WBC) count. Computed tomography (CT) with contrast of the neck revealed soft tissue stranding around the parotid gland. The patient was diagnosed with herpes zoster ophthalmicus (HZO) with concurrent ipsilateral parotitis and subsequently treated with valacyclovir, ofloxacin eye drops, topical erythromycin ointment and amoxicillin/clavulanic acid. Upon follow-up ten days after discharge, the patient noted marked improvement in her symptoms and reduction in pain. To our knowledge, this is the first case described in medical literature of a female patient with HZO and ipsilateral parotitis.

**Topics:**

Herpes zoster opthalmicus, varicella-zoster virus, parotitis.

**Figure f1-jetem-8-2-v6:**
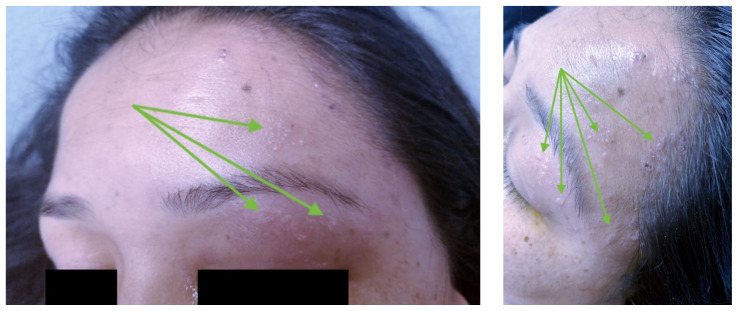


**Figure f2-jetem-8-2-v6:**
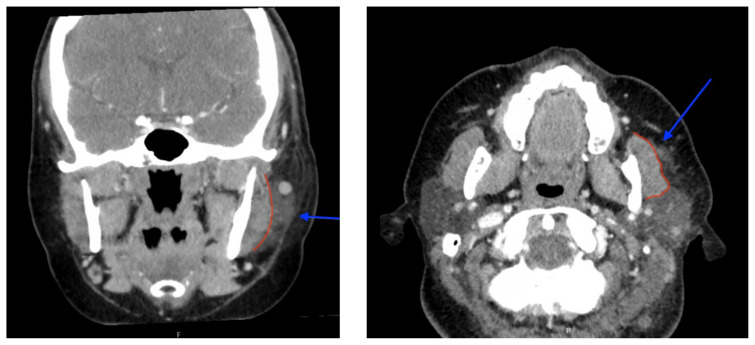


## Brief introduction

[Fig f1-jetem-8-2-v6][Fig f2-jetem-8-2-v6]Varicella–zoster virus (VZV) is a common and highly contagious infection that causes chicken pox in childhood and herpes zoster (HZ), or shingles, later in life.^1^ There are an estimated one million cases of herpes zoster in the US each year.^2^ The virus may remain dormant in the nervous system and reactivate during infections or other immunocompromised states. Herpes Zoster Virus (HZV) initially presents with symptoms of paresthesia, hyperesthesia, dysesthesia and/or pruritus appearing in a dermatomal distribution on the skin. However, clinical symptoms vary and can also include headache, photophobia, fever, and malaise. One day to several weeks later, characteristic skin lesions appear in a dermatomal pattern, beginning as a maculopapular rash and progressing into painful vesicles with an erythematous base.^1^

When VZV is reactivated within the ophthalmic branch of the trigeminal nerve, it causes the condition herpes zoster ophthalmicus.^3^ Patients often present with a unilateral, painful periorbital vesicular rash along the affected facial dermatome. Some also develop keratitis, conjunctivitis, and ocular cranial nerve palsies.^4^ If left untreated, herpes zoster ophthalmicus may lead to chronic ocular inflammation, scarring, and loss of vision.^5^

Inflammation of the parotid gland, termed parotitis, can result from a variety of conditions, the most frequent being viral infections such as mumps.^6^ The appearance of parotitis in cases of varicella zoster virus is extremely rare. We present here a case of parotitis with ipsilateral herpes zoster ophthalmicus in a 36-year-old female. To our knowledge, this is the first presentation of a female patient with parotitis and ipsilateral HZO to be described in medical literature. There have been three previously reported cases, and in all of them the patient was male. Documenting the varied ways in which these two conditions can present, and the different patient populations they may appear in, is of extreme importance. Inclusion of this coinfection in the differential and awareness of its clinical progression may inform future ED protocols and improve the timeliness and efficacy of diagnosis and patient care.

## Presenting concerns and clinical findings

In this case report, a 36-year-old female with a past medical history of hypertension and obesity presented to the emergency department (ED) with five days of headache, left-sided facial pain, and pain in her left eye and left ear. She described first noticing that her left ear was throbbing and that her eye became red and inflamed, feeling as if “something was in her eye.” She reported she normally wears contacts but had not used them since her symptoms began. One to two days later, she began to have a headache behind the left eye, as well as tenderness behind the left ear. At this time, she developed a rash in the left frontal region of her face. The patient reported a similar episode of a rash in the past and was diagnosed with herpes simplex virus (HSV), although she admits she has not seen a primary care physician in over 14 years. The patient denied any vision changes, recent eye surgeries or injections, or recent trauma.

Her initial vital signs included a mildly elevated blood pressure of 155/106, a pulse of 91, a temperature of 98.3°F, a respiratory rate of 18, and blood oxygen saturation of 100% on room air.

The physical exam was remarkable for conjunctival injection of the left eye with multiple vesicular lesions on an erythematous base along the V1 distribution involving the upper and lower eyelids. Fluorescein staining of the eyes revealed pseudodendrites on the left eye with an approximately one by one millimeter defect in the corneal epithelium. Intraocular pressures were normal bilaterally. The patient was also tender to palpation of the left parotid gland and posterior auricular region, as well as over the left mastoid process. The remainder of her exam was unremarkable, including oropharynx, dentition, nares, tympanic membranes and external auditory canals, as well as her neck. Her neurologic exam was also within normal limits.

Given the history and physical of this patient, the differential diagnosis was included but not limited to shingles, mastoiditis, conjunctivitis, corneal abrasion versus keratitis, corneal ulcer, contact dermatitis, and Ramsay Hunt syndrome. Written consent was obtained from the patient for use of images in a case report.

## Significant findings

The patient’s work-up in the emergency department included labs, a computed tomography (CT) scan of the neck, as well as an ophthalmology consult. The patient’s labs were remarkable for a normal white blood cell count of 6.3, as well as a mild elevation of c-reactive protein (CRP) of 1.6 and erythrocyte sedimentation rate (ESR) of 26. The CT soft tissue scan of the neck with intravenous (IV) contrast was remarkable for left deep cervical soft tissue stranding (see blue arrow) predominately centered about the parotid gland (see red outline), which may reflect parotitis and/or cellulitis with likely reactive lymph nodes.

To understand these radiology findings, it is important to comprehend the terminology used by radiologists and how infected tissues are described. Contrast-enhanced CT provides visualization of inflamed and infected tissues since they appear brighter compared to surrounding structures. Radiologists use specific terminology to comment on this relative increase in brightness, describing the tissue as “increased attenuation” or “enhancement.” Additionally, soft tissue stranding describes a change in the attenuation of fat around an inflamed structure and suggests a local infectious or inflammatory process. 7 Classically, uncomplicated cellulitis on CT imaging demonstrates skin thickening, septation of the subcutaneous fat, and thickening of the underlying superficial facsia.^8^ Once the infection spreads to deeper tissues, various tissues can be affected, and the location of that inflammation helps determine the diagnosis. For example, as a cellulitis infection spreads, an abscess can form and reveal a low attenuating necrotic center with a well-defined peripheral fibrous capsule that can be ring enhancing with surrounding fat stranding.^9^ If the deeper layers such as muscle, fascia, or bone become infected, the diagnosis of myositis, necrotizing fasciitis, and osteomyelitis become apparent. Fasciitis, for instance, will show asymmetrical fascial thickening and enhancement associated with fat stranding and edema that may extend into surrounding muscle. 10,11 Local infection also leads to inflammation of nearby lymph nodes, which become enlarged as they attempt to fight the infection.

In our case, the presence of soft tissue stranding about the parotid gland suggested an underlying inflammatory or infectious process of the parotid gland. Cellulitis was considered as a possible diagnosis as well, given the presence of soft tissue stranding in the dermis that is adjacent to the parotid gland. Fortunately, no enhancement was seen in local muscles, fascia, or bones to suggest a myositis, fasciitis, or osteomyelitis. By using the anatomy of the patient and understanding the changes that occur on CT when inflammation is present, the appropriate depth and location of infection can be made, allowing for appropriate treatment regimens.

## Patient course

Ophthalmology was consulted, and the patient was diagnosed with herpes zoster ophthalmicus (HZO) of the left eye with an associated zoster distribution along V1, as well as conjunctival and corneal abrasion of the left eye. Ophthalmology recommended initiating valacyclovir one gram three times a day for ten days, ofloxacin drops four times a day to the left eye, as well as topical erythromycin ointment to the skin lesions and eyelids twice daily. Preservative free artificial tears (PFAT) were also recommended four to six times a day applied to both eyes, as well as warm compresses. Ophthalmology also recommended an otolaryngology consult given the associated ear pain in the setting of VZV. Otolaryngology recommended oral and intravenous hydration as well as oral antibiotics to cover for *staphylococcus* and *streptococcus species*, such as amoxicillin/clavulanic acid or clindamycin for the parotitis. Additional recommendations for the parotitis included sialagogues to stimulate saliva production, as well as warm compresses every hour for thirty minutes with parotid massage. The patient was discharged after staying in the emergency department observation unit overnight to receive the consultation recommendations.

Outpatient follow up ten days after discharge with otolaryngology revealed complete resolution of the patient’s pain. She reported significant improvement to her cheek swelling and denied ever experiencing visual deficits. She remained compliant with her medications, sialagogues, warm compresses, and parotid massages. Follow-up with ophthalmology three weeks after discharge noted improvement in her condition as well after completing her treatments and no additional follow up was required.

## Discussion

Herpes zoster ophthalmicus is a viral infection caused by reactivation of the varicella zoster virus, which can lead to severe ocular complications such as blindness if left untreated. Concurrent ipsilateral parotitis, or inflammation of the parotid gland on the same side as the ocular involvement, is a rare complication of HZO. Fortunately, both HZO and parotitis are easily diagnosed with standard emergency department resources, which include a physical exam showing vesicles and ulcerations (green arrows in the images) in a dermatomal distribution, fluorescein staining with pseudodendrites, and a CT scanner showing inflammation of the parotid gland. Although our academic tertiary care center can consult Ophthalmology and Otolaryngology for these cases, it is not required in community emergency medicine and does not preclude appropriate outpatient management of patients with this diagnosis.

One limitation of the case was the lack of clear treatment guidelines that currently exist, given the rarity of this coinfection. Interestingly, the case presented in this report is, to our knowledge, the first female patient with HZO and concurrent ipsilateral parotitis described in medical literature. At age 36, this patient is also the youngest to be discussed since the three previous cases of this coinfection were in male patients ages 38, 43, and 58.^12^,^13^,^14^ There are several key similarities between all four reported cases to date: all patients were immunocompetent, they presented afebrile, and they had numbness, swelling, and a vesicular rash.^12^,^13^,^14^ Additionally, all four cases involved ipsilateral coinfections, although the side of the face involved varied. Treatment regimens, however, varied from case to case, and included prednisone and indomethacin,^12^ acyclovir,^13^ or valacyclovir with amoxicillin/clavulanate and prednisone.^14^ We chose to treat our patient’s infection with valacyclovir, ofloxacin eye drops and topical erythromycin ointment, as well as amoxicillin/clavulanate for the parotitis.

The mechanism of connection underlying these two coinfections remains unknown. Yoshida et al proposed the reactivated VZV can possibly travel from the trigeminal nerve through the auriculotemporal nerve to the parotid gland.^14^ However, Banno et al proposed that the parotitis caused an underlying immune dysregulation in the patient that enabled the VZV to reactivate.^12^ Regardless of the mechanism, our patient’s unique presentation highlights the importance of recognizing the varying ways in which VZV can present itself because involvement may extend beyond the eye and into external structures, as it did here. It is crucial for healthcare providers to have a high level of suspicion for herpes zoster ophthalmicus in patients with ocular symptoms and to consider the possibility of concurrent ipsilateral parotitis. Early diagnosis and treatment are essential in preventing significant harm to the eye and preserving visual acuity.

## Supplementary Information
















