# Supplementary figures and images for: Case Report of Herpes Zoster Ophthalmicus with Concurrent Parotitis
Source: J Educ Teach Emerg Med. 2023 Apr 30;8(2):V6–V10. doi: 10.21980/J8R93N (PMC10332680; doi:10.21980/J8R93N)

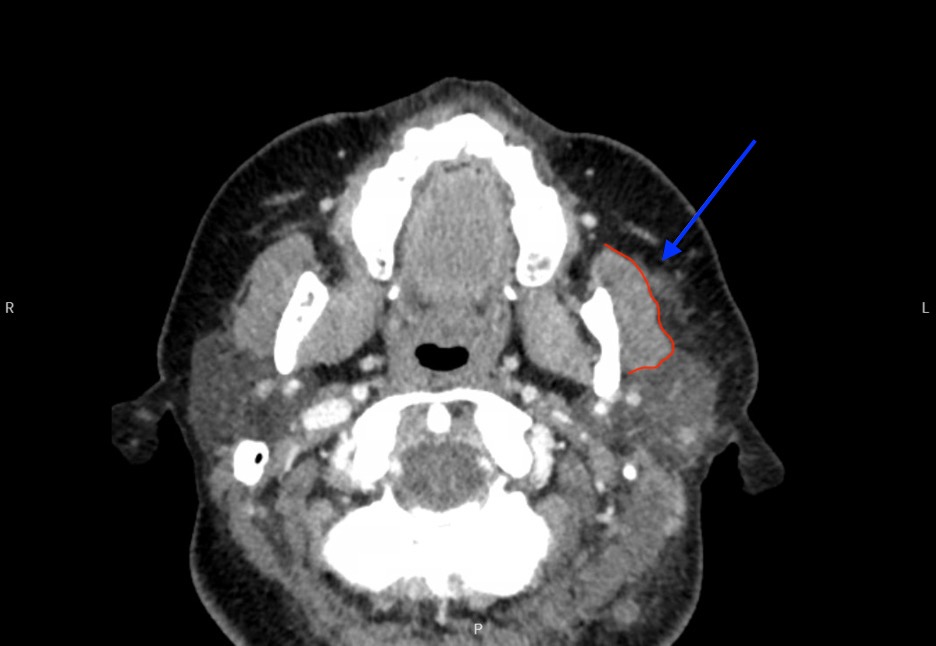

Supplement: Supplementary file 1 [file JETem-8-2-V6-supp1.jpg]

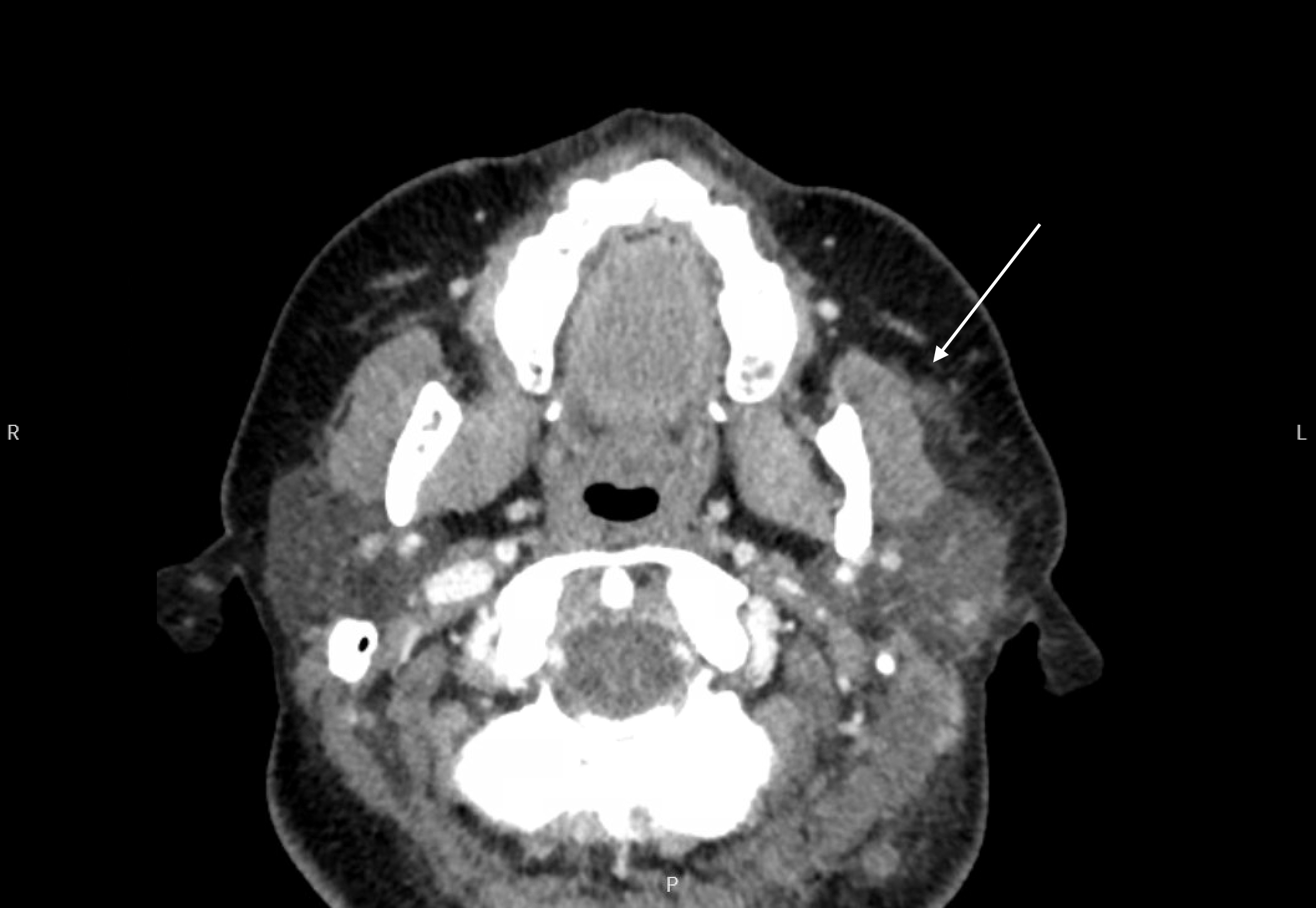

Supplement: Supplementary file 2 [file JETem-8-2-V6-supp2.jpg]

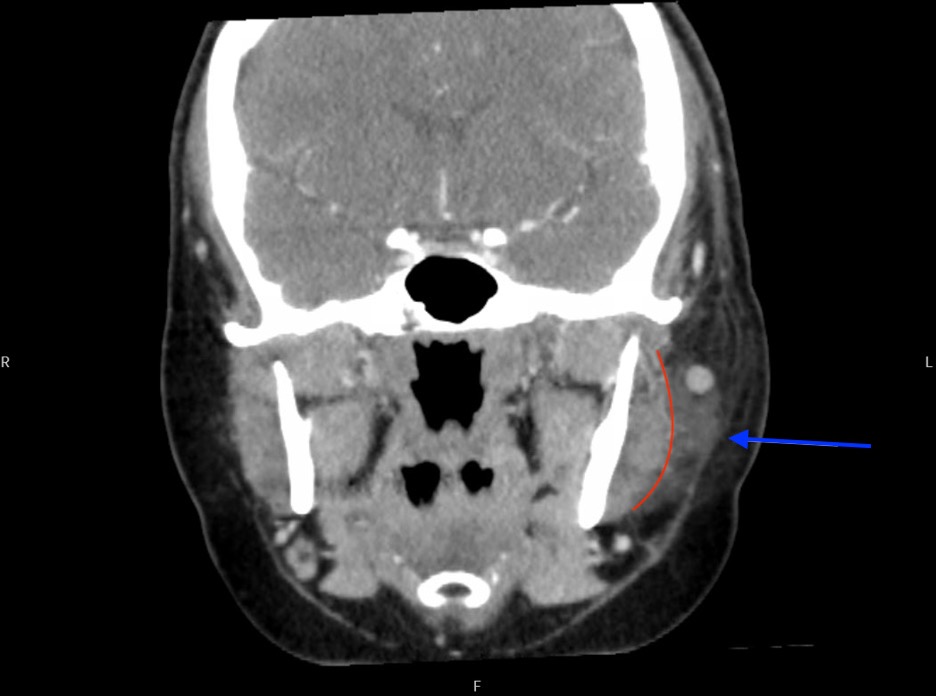

Supplement: Supplementary file 3 [file JETem-8-2-V6-supp3.jpg]

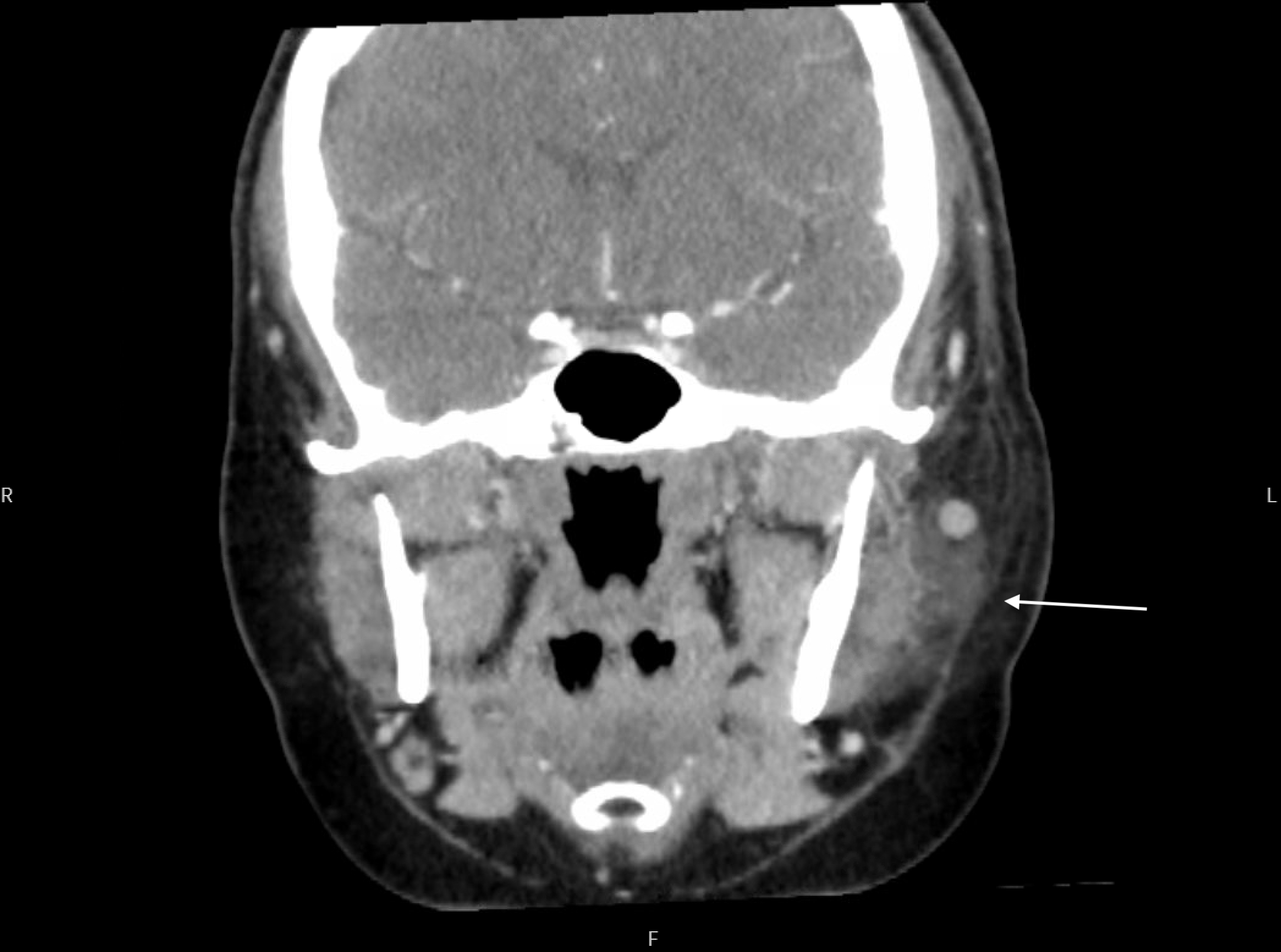

Supplement: Supplementary file 4 [file JETem-8-2-V6-supp4.jpeg]

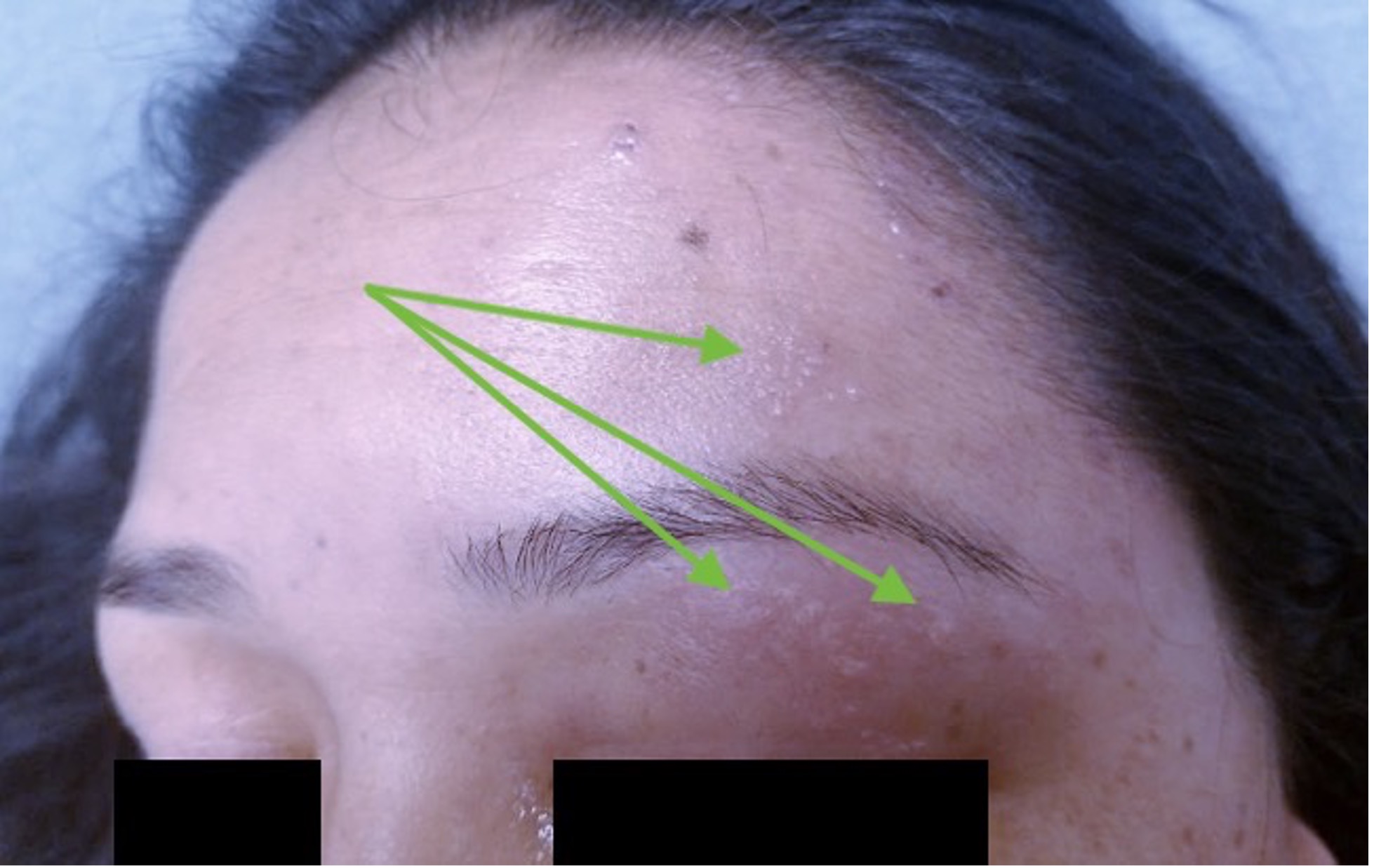

Supplement: Supplementary file 5 [file JETem-8-2-V6-supp5.jpg]

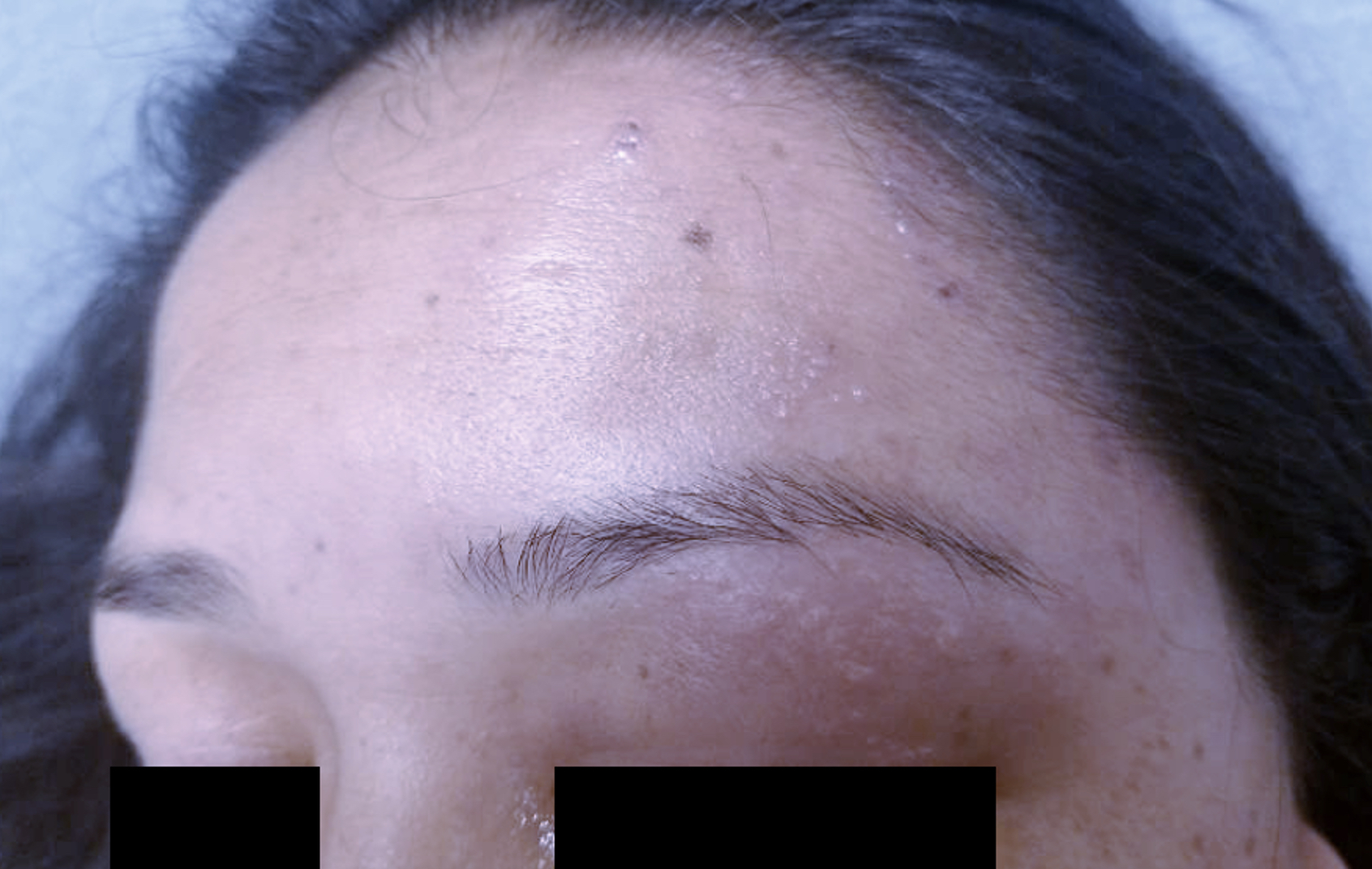

Supplement: Supplementary file 6 [file JETem-8-2-V6-supp6.jpeg]

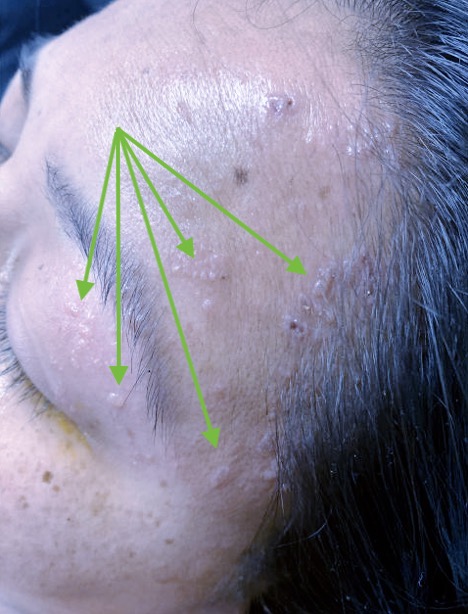

Supplement: Supplementary file 7 [file JETem-8-2-V6-supp7.jpg]

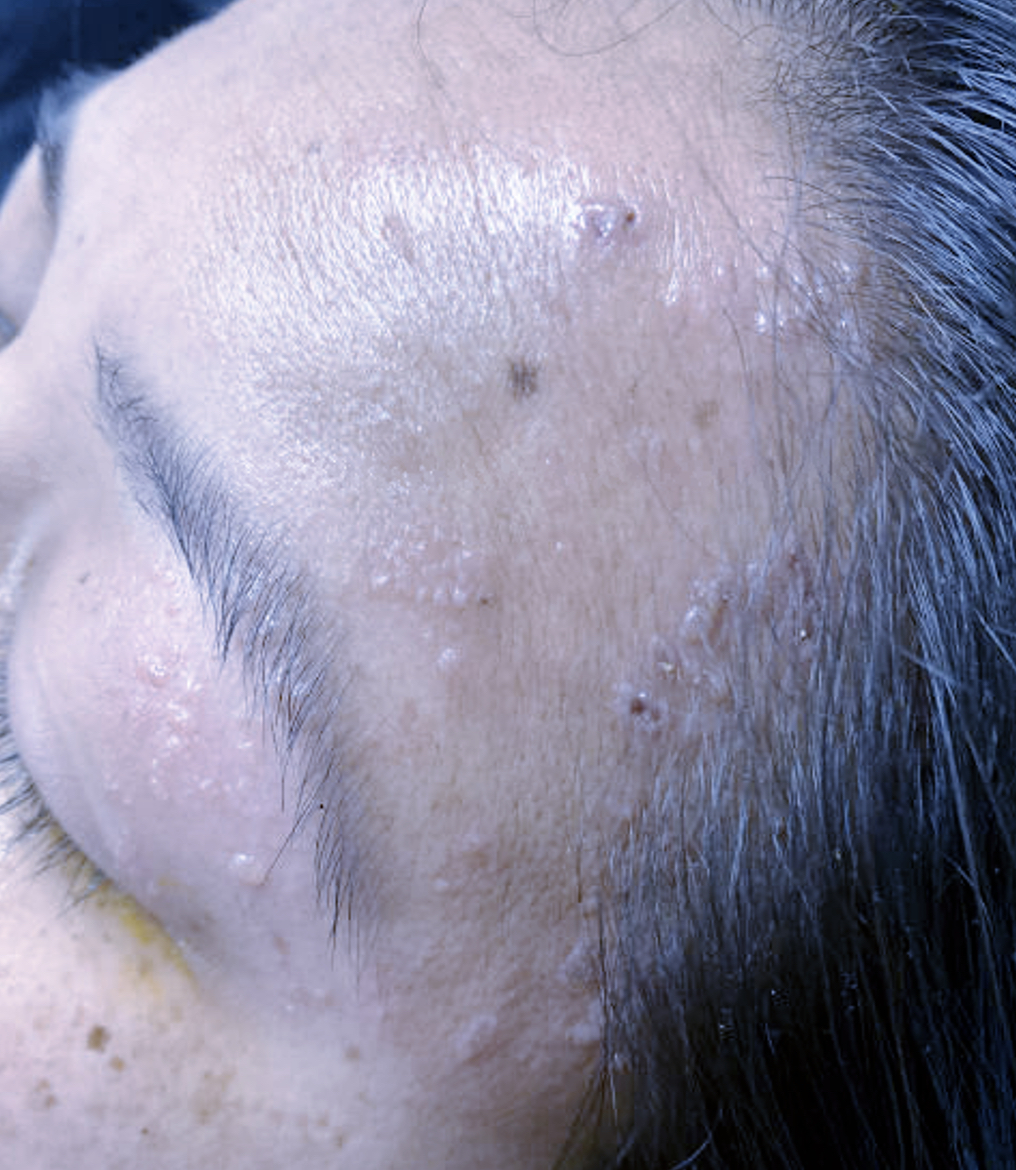

Supplement: Supplementary file 8 [file JETem-8-2-V6-supp8.jpeg]
